# Sick leave, disability, and mortality in acute hepatic porphyria: a nationwide cohort study

**DOI:** 10.1186/s13023-019-1273-4

**Published:** 2020-02-21

**Authors:** Carl Michael Baravelli, Aasne Karine Aarsand, Sverre Sandberg, Mette Christophersen Tollånes

**Affiliations:** 1 Norwegian Porphyria Centre (NAPOS), Department of Medical Biochemistry and Pharmacology, Haukeland University Hospital, P.O.Box 1400, N-5021 Bergen, Norway; 2 Department of Global Public Health and Primary Care, University of Bergen, Bergen, Norway; 3 Norwegian Organisation for Quality Improvement of Laboratory Examinations (NOKLUS), Haraldsplass Deaconess Hospital, Bergen, Norway

**Keywords:** Acute hepatic porphyria, Acute intermittent porphyria, Variegate porphyria, Hereditary coproporphyria, Long-term sick leave, Disability pension, Morbidity, Premature death, Mortality

## Abstract

**Background:**

Acute hepatic porphyria (AHP) consists of three rare metabolic disorders. We investigated the risk of long-term sick leave, disability pension, and premature death in individuals with AHP compared to the general population.

**Methods:**

In a nationwide cohort study from 1992 to 2017, records of 333 persons (total person-years = 6728) with a confirmed AHP diagnosis were linked to several national compulsory registries (reference populatio*n* = 5,819,937). We conducted survival analyses to assess additional risk.

**Results:**

Persons with AHP had higher risks of accessing long-term sick leave (adjusted hazard ratio (aHR): 1.5, 95% confidence interval (CI): 1.3, 1.7) and disability pension (aHR: 1.9, CI: 1.5, 2.4). The risk was highest in persons who had been hospitalised for acute attacks, while no additional risk was observed in asymptomatic AHP gene mutation carriers. The median age when accessing disability pension was 45 years, 21 years younger than the general population. AHP was associated with increased risk of mortality due to hepatocellular carcinoma (adjusted mortality rate ratio (aMRR): 84.4, CI: 37.8, 188.2), but no overall increased risk of premature death was observed.

**Conclusions:**

Persons with symptomatic AHP were at increased risk of accessing long-term sick leave and disability pension but not of premature death.

## Background

Autosomal dominant acute hepatic porphyria (AHP) refers to three rare metabolic disorders that affect the biosynthesis of haem, namely acute intermittent porphyria (AIP), variegate porphyria (VP) and hereditary coproporphyria (HCP). All can present clinically in the form of acute neurovisceral attacks characterized by neuropathic pain, mostly abdominal, and may be accompanied by muscular pain, nausea, vomiting, constipation, general malaise, fatigue, psychiatric and neurological symptoms [1]. Acute attacks typically do not occur until adulthood and are more common in women [1]. However, the clinical presentation is highly variable. If left untreated, attacks can result in seizures, paralysis, and, in very rare situations, death [2]. VP and HCP may present with solely cutaneous lesions or both acute and cutaneous symptoms.

AHP is characterized by the accumulation of porphyrin precursors 5-aminolevulinic acide (ALA) and porphobilinogen (PBG), and in VP and HCP, porphyrins [3]. ALA and PBG concentrations are increased during acute attacks [4]. Symptomatic AIP has an estimated prevalence in Norway of seven in 100,000 persons [5]. In European countries, the prevalence of VP and HCP are estimated at one in 30,000 and less than one in 50,000, respectively [6]. However, clinical penetrance is incomplete, with many genetically predisposed never having symptoms. The overall prevalence of clinically relevant AIP gene mutations may be as high as ~ 6/1000 among caucasians [7].

Persons with symptomatic AHP report low health-related quality of life [8–10] and high access rates to long-term sick leave and disability pension [1, 11]. Persons with more severe recurrent acute attacks further report debilitating chronic symptoms between attacks, such as chronic pain, fatigue and aspects of neuropathy [10–13], as well as high levels of unemployment [14]. However, it is difficult to determine if such outcomes were increased compared to the general population or may have been confounded by, for instance, age, sex, or socio-economic factors. Long-term complications of AHP, in particular in AIP, include life-threatening diseases such as kidney failure [15], hypertension [16] and hepatocellular carcinoma (HCC), with the latter typically presenting in the absence of cirrhosis and other risk factors [17, 18].

In our study, we aimed to investigate if persons with AHP were at increased risk of long-term sick leave, disability pension, and premature death compared to the general population and if there were any differences in risk between symptomatic and asymptomatic AHP gene mutation carriers.

## Methods

### Data sources

The Norwegian Porphyria Centre (NAPOS) was established in 1999 and maintains an administrative database system of all persons with either symptomatic or a genetic predisposition for AHP across Norway. Records include the AHP diagnosis, date of diagnosis and biochemical and genetic laboratory test results. Additionally, all such persons are invited to participate in the Norwegian Porphyria Registry, a national medical quality registry, which was established in 2002 and is administered by NAPOS [19]. Data for the registry are mostly derived from patient-reported questionnaires supplemented with laboratory test results. Participants completed questionnaires two years following the initial submission, and thereafter, every four years. The registry is based on patient consent with an overall participation rate of 71% and an average response rate to follow-up patient-reported questionnaires of 73%. Porphyria diagnosis is confirmed either by biochemical testing and/or DNA analysis, conducted by the Department of Medical Biochemistry and Pharmacology and Centre for Medical Genetics and Molecular Medicine, Haukeland University Hospital.

The National Registry contains demographic information of all Norwegian residents since 1876 and is administered by the Norwegian Tax Administration [20]. The National Education Database maintains individual-based education statistics for all residents of Norway from primary to tertiary level and is administered by Statistics Norway.

The Norwegian Labour and Welfare Administration has maintained records regarding disbursements of various benefits, including long-term sick leave benefit and disability pension, since 1992 [21]. To qualify for disability pension, a person has to be aged 18 years or older and have a permanently reduced earning capacity by 50% or more due to illness or injury. In Norway, the first 16 calendar days of a sick leave episode are compensated by the employer. Therefore, data for sick leave episodes that lasted less than 17 days were not available.

Medical doctors complete a death certificate for all deaths, which is recorded in the Cause of Death Registry of Norway. The registry’s degree of coverage is higher than 98% [22].

Using the unique national identification number assigned to each Norwegian at birth (or immigration), precise person-level record linkage was performed between the data-sources.

### Study design and study population

**Fig. 1:**
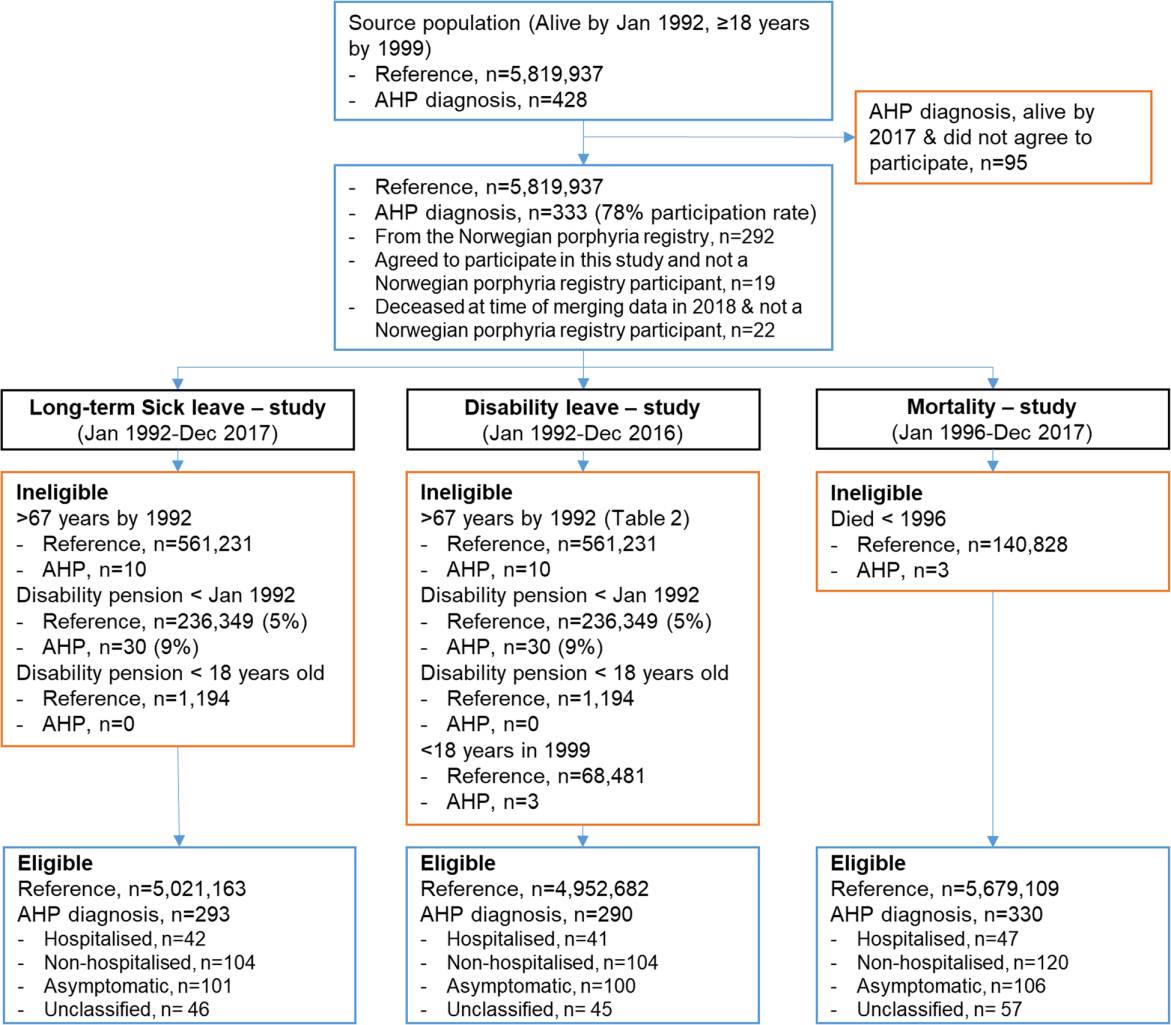
Overview of eligibility criteria and study sample

We conducted a population based, nationwide, cohort study using registry data. All Norwegian residents registered in the National Registry, alive at the study start in January 1992 or born during 1992–1999, were included in our initial cohort. The reference population comprised of 5,819,937 adults in the initial cohort. Of the 428 persons in Norway with a confirmed diagnosis of symptomatic AHP or a predictively tested asymptomatic AHP gene mutation carrier, hereon referred to as asymptomatic AHP gene mutation carriers, 333 persons participated in the current study (participation rate = 78%) (AIP = 292; VP = 32; HCP = 9). They consisted of 292 participants from the consent based Norwegian Porphyria Registry, 19 additional persons from the NAPOS administrative database system who were invited and gave written consent to participate in the current study, and 22 persons from the NAPOS administrative database system who were deceased at the time of planning the study. The study period differed according to the availability of the outcome datasets. Additionally, when investigating long-term sick leave and disability pension, persons who had received a disability pension before the study start in January 1992 or who were 67 years of age or older were excluded. Eligibility criteria and Cohort sizes according to the three outcomes are displayed in Fig. 1.

We classified persons with AHP into four sub-groups: 1) ‘Hospitalised AHP’, persons who reported having been hospitalised at least once due to an acute attack’; 2) ‘Non-hospitalised AHP’, persons who reported having had symptoms of porphyria but never having been explicitly hospitalised for an acute attack; 3) ‘Asymptomatic AHP gene mutation carriers’, persons who reported never having had symptoms of porphyria; and 4) ‘Unclassified’, persons with a confirmed AHP diagnosis but who had not participated in the Norwegian Porphyria Registry and therefore had not answered clinically relevant questions.

Selected disease diagnoses and codes for the three outcome measures are listed in Table 1.

**Table 1:** Diagnostic codes investigated

**Long-term sick leave diagnoses**	**ICPC-2 code**
General and unspecified	A01-A99
Weakness/ tiredness general	A04
Abdominal pain	D01-D02, D06
Cardiovascular	K01-K99
High blood pressure/hypertensive disorder	K85, K86, K87
Ischemic heart disease	K76
Muscle/joint – pain/symptoms	L18, L19, L20
Neurological	N01-N99
Psychological	P01-P99
Acute stress reaction	P02
Feeling depressed	P03
Depressive disorder	P76
Endocrine/ metabolism/ nutritional disorder, other	T99
Urological symptoms and diseases	U01-U99
**Disability pension diagnoses**	**ICD-10 code**
Neoplasms	C00–96, D45–47
Disorders of porphyrin and bilirubin metabolism	E80
Mental and behavioral disorders	F00-F99
Epilepsy	G40
Diseases of the circulatory system	I00-I99
Hypertension	I10-I15
Ischemic heart disease	I20-I25
Diseases of the musculoskeletal system and connective tissue	M00-M99
Renal failure	N17-N19
**Causes of death diagnosis (underlying and contributing)**	**ICD-10 code**
Malignant neoplasms	C00–96, D45–47
Hepatocellular carcinoma	C22.0
Renal carcinoma	C64
Type I diabetes	E10
Hypertension	I10-I11
Ischemic heart disease	I20-I25
Renal failure	N17-N19

Abbreviations: *ICPC-2* International Classification of Primary Care – 2nd edition; *ICD-10* International Classification of Diseases (ICD) – 10th revision

### Statistical analysis

Stata/SE Version 15 for Windows was used for all statistical analyses (Software: Release 15, College Station, TX, USA). We calculated annual incidence rates by dividing the number of new cases by 100 person-years. We estimated the hazard ratio (HR) and corresponding 95% confidence intervals (CIs) to assess the risk of accessing long-term sick leave, disability pension or premature death in persons with AHP compared to the general population, by Cox proportional hazard regression models. Age on the study (months, years) was the time-scale for the analyses. Porphyria diagnosis (0 = reference population, 1 = AHP diagnosis) or AHP sub-groups (0 = reference population, 1 = hospitalised AHP, 2 = non-hospitalised AHP, 3 = asymptomatic, 4 = unclassified) were entered as exposure variables. The entry time was the start of the study, or, if younger than 18 years at the study start, the month and year of the participant’s respective 18th birthday. The exit time was the month and year of the outcome of interest, death, or the end of the study, whichever came first. Additionally, persons exited the study at the retirement age of 67 years when assessing sick leave and disability pension. When assessing sick leave benefits, we censored for the date of disability leave. The Cox regression analyses were stratified by birth cohorts of 20 years, to adjust for calendar effects. We performed crude analyses, adjusting for age as the time-scale. Further, we ran models adjusting for sex, and the level of educational attainment (no education, primary and middle school education (1 to 10 years), intermediate education (11, 12, 13) tertiary education (14 years or more), and unspecified). We produced non-parametric hazard estimate curves with 95% CIs to visually display risk. Tests of interactions between AHP diagnosis and sex were conducted by including a product term in each model. We also investigated if the highest ever recorded concentration of urinary PBG, or ALA (ALA/PBG by Column Test, Bio-Rad Diagnostics) predicted the risk of accessing disability pension in separate Cox proportional hazard models. The proportionality assumption of the Cox models was assessed by inspecting Kaplan-Meier curves and the log(−log (survival)) versus log (time) graphs for fixed covariates, including time-dependent covariates in the model for all covariates, and tests of the non-zero slope. No violations were detected.

To assess differences in diagnostic reasons for long-term sick leave and disability pension between persons with AHP and the general population, we conducted Poisson regression analyses with robust standard errors to estimate the incident rate ratios (IRR) and CIs, offset for months on study (month and year of exit minus month and year of entry).

We performed all analyses in a traditional cohort design, using the reference population as controls. Additionally, given the differences in age between our groups, we performed matched case-control analyses of the primary outcomes. The matched analysis used ten controls to every case, randomly selected from the population and frequency-matched on sex, age at study start, and educational attainment.

In a sensitivity analysis for mortality, 96 non-participants with an AHP diagnosis, known to be alive by the study end in 2017, were included in crude analyses.

### Ethical approval

The study was approved by the Regional Committees for Medical and Health Research Ethics, Norway (reference number: 2012/753).

## Results

Participants with non-hospitalised and unclassified AHP were generally older at the start of the study compared to the other groups. High proportions of hospitalised and non-hospitalised AHP subjects were female. The unclassified subjects tended to have lower educational attainment (Table 2).

**Table 2:** Baseline characteristics of acute hepatic porphyria sub-groups compared to the reference population (1992 to 2017, 18–67 years of age)

Characteristics	Hospitalised (*n* = 42)		Non-hospitalised (*n* = 104)		Asymptomatic (*n* = 101)		Unclassified (*n* = 46)		Reference population (*n* = 5,021,163)	
	n/ mean	%/(95% CI)	n / mean	%/(95% CI)	n / mean	%/(95% CI)	n / mean	%/(95% CI)	n / mean	%/(95% CI)
Sex – female	29	69.1	63	60.6	48	47.2	25	54.4	2,427,746	48.4
Age in years (study start)	29.0	(24.9, 33.0)	34.7	(32.2, 37.2)	27.0	(24.6, 29.4)	32.8	(28.7, 36.9)	29.0	(29.0, 29.0)
Education attainment										
Unspecified	0	0.0	0	0.0	0	0.0	0	0.0	636,494	12.7
Primary/middle edu (1–10 yrs)	8	19.1	22	21.2	20	19.8	17	37.0	1,113,270	22.2
Intermediate edu (11–13 yrs)	18	42.9	42	40.4	44	43.6	19	41.3	1,827,443	36.4
Tertiary edu (14+ yrs)	16	38.1	40	38.5	37	36.6	10	21.7	1,443,956	28.8
Biochemical characteristics										
PBG	24.5	(14.4, 34.5)	11.8	(8.9, 14.7)	4.1	(2.6, 5.5)	7.8	(3.9, 11.7)		
ALA	24.5	(11.3, 37.7)	10.9	(8.6, 13.3)	4.9	(4.0, 5.9)	6.8	(4.0, 9.9)		
PBG > 4*upper reference limit	33	78.6	68	65.4	24	23.8	15	32.6		
PBG > 10*upper reference limit	26	61.9	39	37.5	18	17.8	13	28.3		
Smoking										
Never / have quit	24	57.1	66	63.5	74	73.3				
Occasionally / daily	16	38.1	35	33.7	20	19.8				
Not specified	2	4.8	3	2.9	7	6.9				
Drinks alcohol > 3 / week										
Yes	1	2.4	1	1.0	2	2.0				
No	26	61.9	77	74.0	61	60.4				
Not specified	15	35.7	26	25.0	38	37.6				
Body mass index (BMI)	23.7	(22.5, 24.9)	25.7	(24.8, 26.6)	23.4	(22.5, 24.4)				

Note: *AHP* acute hepatic porphyria; *CI* confidence intervals; *PBG* porphobilinogen; *ALA*: 5-aminolevulinic acid

Lifestyle factors are based on self-reported questionnaires sent to the Norwegian Porphyria Registry. Biochemical characteristics are based on the highest ever recorded value, recorded mostly outside of an acute attack. PBG upper reference limit > 0.8 umol/mmol creatinine; ALA upper reference limit > 4.6 umol/mmol creatinine

### Long-term sick leave

**Fig. 2:**
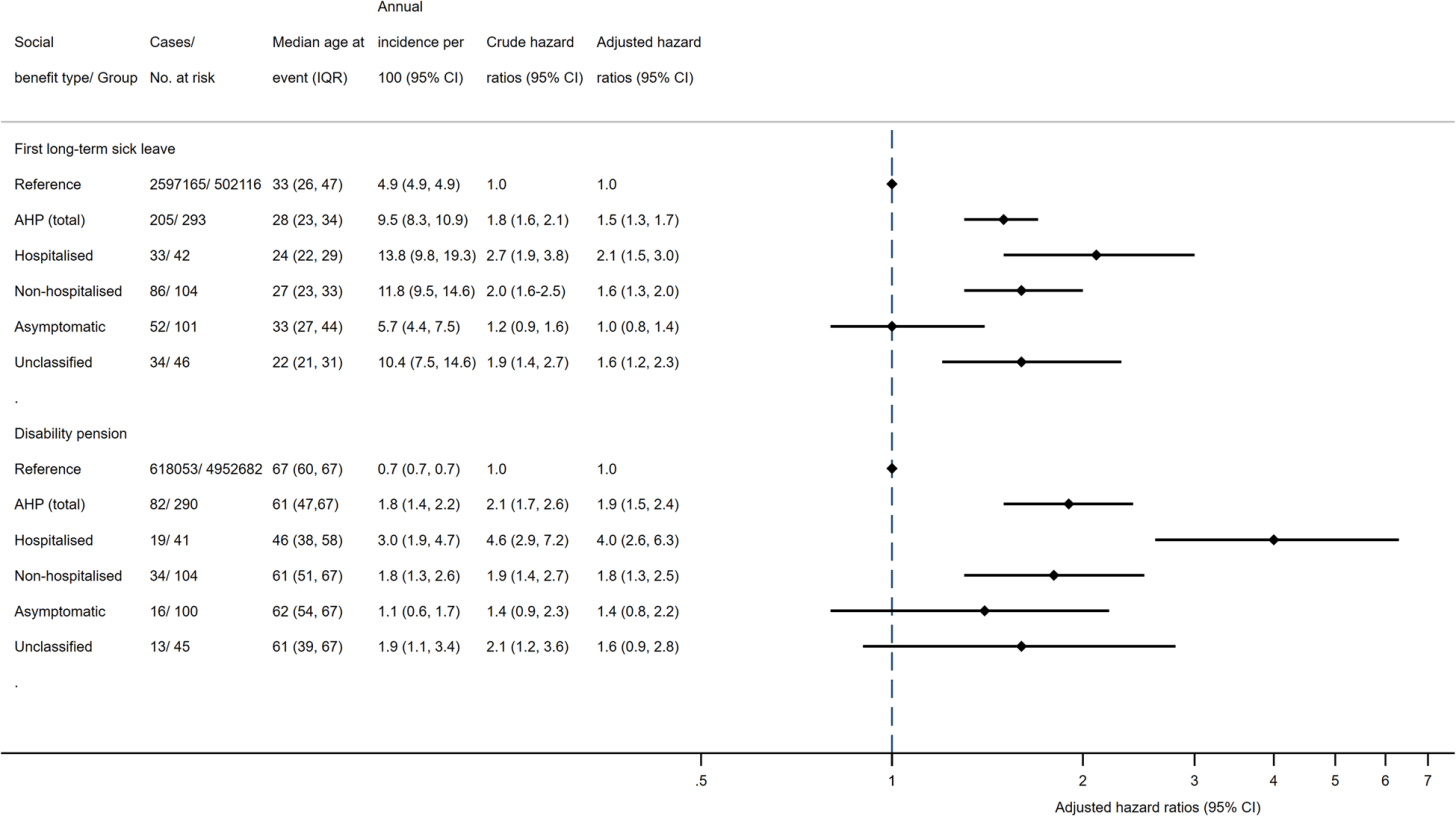
Risk of long-term sick leave (17 days or more) at least once over a lifetime and disability pension in persons with acute hepatic porphyria and the reference population aged 18 to 67 years. Note: IQR: interquartile range (25th, 75th percentiles); AHP: acute hepatic porphyria; CI: Confidence intervals. The X-scale is logarithmic. Adjusted analysis: Adjusted for age in years (timescale), sex and educational attainment and stratified by birth cohorts.

**Fig. 3:**
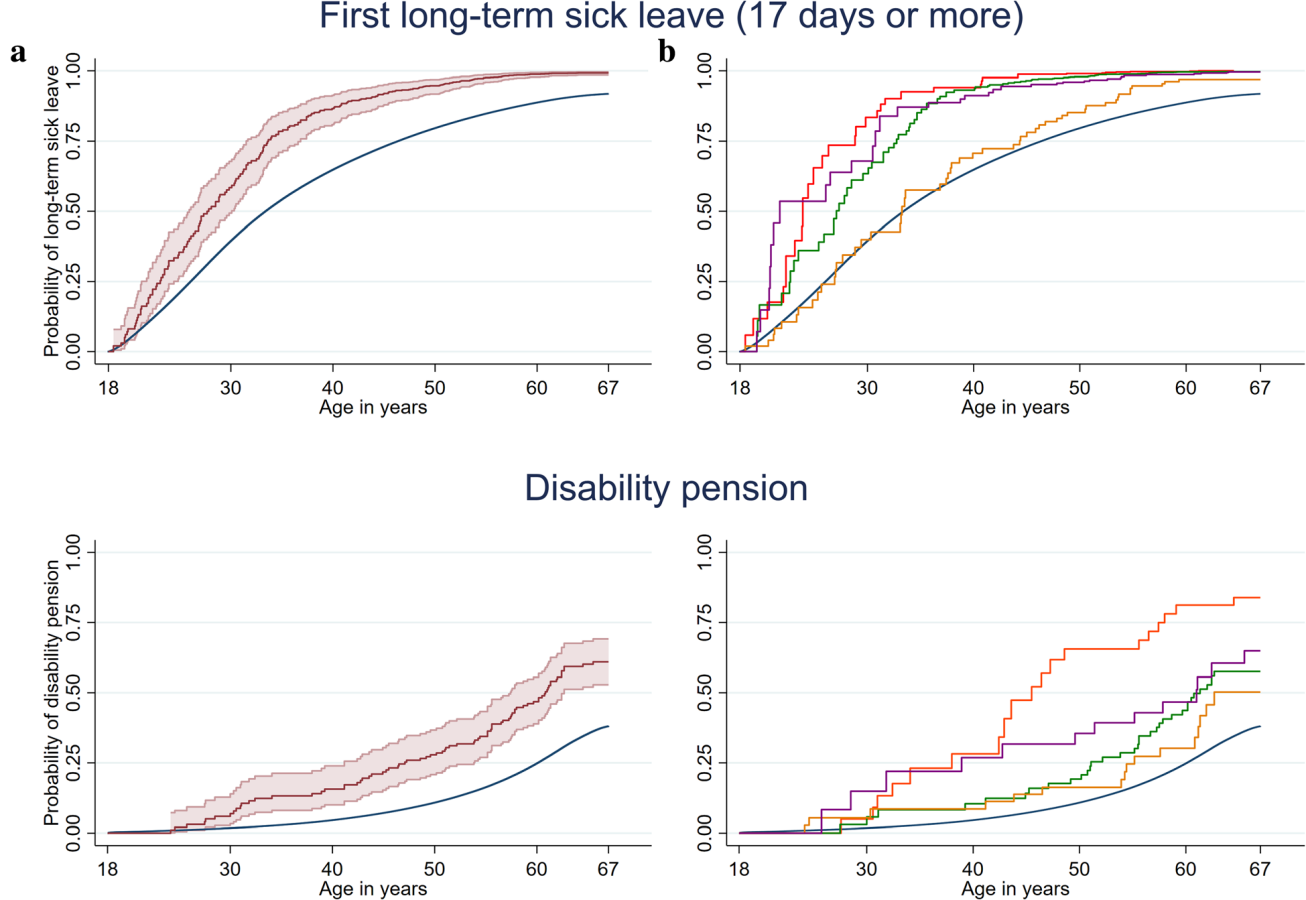
Kaplan-Meier curve for cumulative failure probability estimates of first long-term sick leave event and disability pension from 18 to 67 years of age between persons with acute hepatic porphyria (red line, panel **a**) and sub groups (red line=hospitalised, green line=non-hospitalised, yellow line=asymptomatic, purple line=unclassified, panel **b**) and the reference population (blue lines, panel a and b). Note: AHP: acute hepatic porphyria; CI: confidence intervals; 95% confidence intervals displayed on panel A only; 95% confidence intervals in panel A of the reference are too small to detect

Overall, 70% of persons with AHP accessed long-term sick leave throughout the study period compared to 52% of the general population, constituting an annual incidence of 9.5% and an HR of 1.5 (95% CI: 1.3, 1.7) (Figs. 1 & 2). The risk was highest in persons with a history of a hospitalised acute attack (HR = 2.1, 95% CI: 1.5, 3.0), while not elevated in asymptomatic AHP gene mutation carriers (HR = 1.0, 95% CI: 0.8, 1.4) (Figs. 2 & 3). Persons with AHP were, on average, 5 years younger at the time of their first long-term sick leave episode than the reference population.

**Fig. 4:**
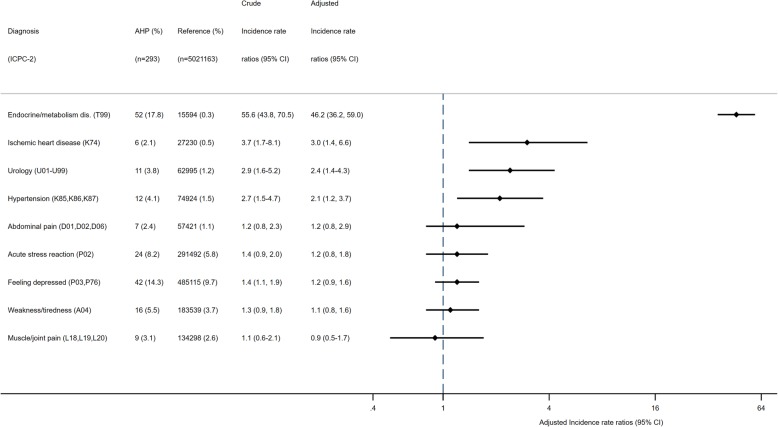
Diagnostic reasons for a long-term sick leave episode between persons with AHP acute hepatic porphyria and sub-groups and the reference population (aged 18 to 67 years). Note: IRR = Incident rate ratios; CI=Confidence intervals; dis = disorder. The X-scale is logarithmic. Adjusted analysis: Adjusted for age in years, sex and educational attainment and stratified by birth cohorts.

The main diagnostic reason for long-term sick leave in patients with AHP was ‘endocrine/metabolism/nutritional disorder’ (*n* = 52 patients), which includes an AHP diagnosis (Fig. 4). Psychological symptoms/disorders were also common reasons for long-term sick leave both in AHP and the general population (Fig. 4). Compared to the general population, individuals with an AHP diagnosis had an increased risk of a long-term sick leave episode due to high blood pressure, ischemic heart disease, endocrine/metabolism/nutritional disorder, and a urological symptom/disorder (Fig. 4).

### Disability pension

Having an AHP diagnosis resulted in a 1.9-fold (95% CI: 1.5, 2.4) increased risk of accessing disability pension compared to the general population (Figs. 2 & 3). The risk was highest in persons with hospitalised AHP (HR = 4.0, 95% CI: 2.6, 6.3), who were, on average, 21 years younger than the general population when accessing disability pension (Figs. 2 & 3). No trend between highest ever recorded urinary PBG (*p* = .246) and ALA (*p* = .327) concentrations and risk of DP was detected in persons with AHP. The risk of accessing disability pension in AHP did not depend on sex (*p*-value for interaction = .919). Of those on a disability pension with an AHP diagnosis, 70 persons had AIP (27% of AIP cases), 8 VP (31% of VP cases) and 4 HCP (44% of HCP cases).

The most common diagnostic reason persons with AHP were on a disability pension was because of their AHP (ICD-10: E80.2), either as a primary diagnosis (*n* = 16) or secondary diagnosis (*n* = 9). Of the 16 individuals who received a disability pension because of their AHP, six had hospitalised AHP, nine had non-hospitalised AHP, and one was unclassified. Other diagnostic reasons for accessing disability pension included mental and behavioral disorders (F00-F99), *n* = 10; diseases of the circulatory system (I00-I99), *n* = 10; and diseases of the musculoskeletal system and connective tissue (M00-M99), *n* = 10. However, the risk was comparable to that of the general population, except for diseases of the circulatory system (aIRR = 3.8, 95% CI: 2.0, 7.1).

### Mortality

**Fig. 5:**
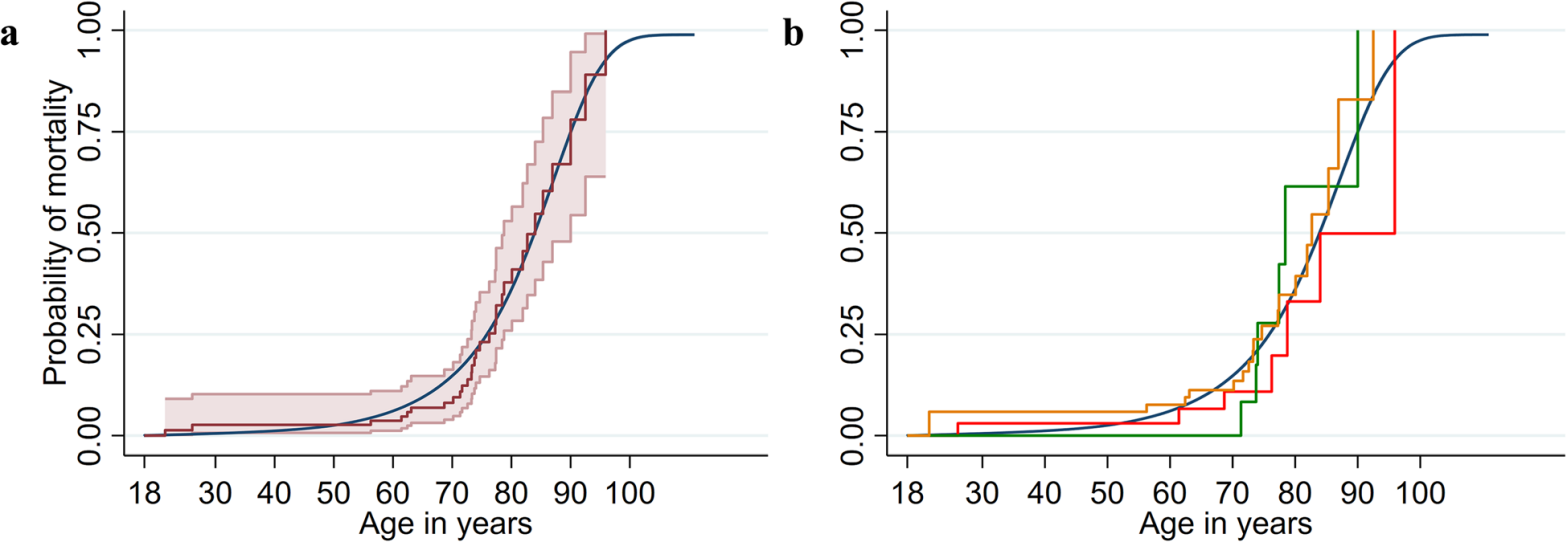
Kaplan-Meier curve for cumulative mortality estimates from 18 to 67 years of age between persons with AHP (red line, panel **a**) and sub groups (red line=hospitalised, green line=non-hospitalised, yellow line=asymptomatic, panel **b**) and the reference population (blue lines). Note: AHP: acute hepatic porphyria; CI: confidence intervals; 95% CIs displayed on panel A only; 95% CIs in panel A of the reference are too small to detect. Excludes persons with unclassified AHP

Persons with AHP had a 1.3-fold (95% CI: 1.0, 1.8) increased risk of premature death compared to the general population. In the sub-groups, risk was only increased in unclassified subjects (aHR = 3.2, 95% CI: 2.1, 4.4), but not in hospitalised AHP subjects (aHR = 1.0, 95% CI: 0.5, 2.5), non-hospitalised AHP subjects (aHR = 1.0, 95% CI: 0.6, 1.6), or asymptomatic AHP gene mutation carriers (aHR = 0.7, 95% CI: 0.3, 1.4). In the sensitivity analysis in which 95 non-participants with a known AHP diagnosis (who were known to be alive by the study end in 2017) were included in crude analysis, no increased risk of premature death was observed (IRR = 0.8, 95% CI: 0.6, 1.0) (Fig. 5). There was no observed difference in median age at death between the total population and persons with AHP or AHP sub-groups.

Nineteen deaths were due to malignancies, six specifically due to HCC carcinoma (ICD-10: C22.0), and five due to ischemic heart disease (ICD-10: I20–25). Compared to the reference population, an increased cause-specific risk of death was observed only for HCC (adjusted mortality rate ratio (aMRR) = 84.4, 95% CI: 37.8, 188.2). In the sensitivity analysis, the aMRR decreased to 58.9 (95% CI: 26.5, 130.5). Other causes of death of interest, but with a count less than three, included: renal carcinoma (C64): *n* = 2; porphyria (E80.2), *n* = 2; and renal failure (N17–19), *n* = 2. There was one additional count of HCC, three counts of hypertensive disorder (I10, I11) and five additional cases of renal failure (N17–19) listed as contributing, but not the main underlying cause of death.

### Matched analysis

The results from the matched analysis were essentially identical to the analysis which used the entire reference population (Additional file 1: Table S1).

## Discussion

AHP is a common term for three porphyria diseases which have highly variable natural histories and clinical presentation in different patients. Some AHP gene mutation carriers remain asymptomatic throughout their lives, some have one to two attacks during their lifetime while others are severely affected, having recurrent acute attacks requiring frequent hospitalizations and low quality of life. Patients with VP and HCP may have symptomatic disease only in the form of skin symptoms. AHP patients with recurrent acute attacks also report chronic symptoms outside of attacks, resulting in increased morbidity. The diseases are in addition associated with several long-term complications that may increase risk of a premature death. In a population based cohort study, we found that having a symptomatic AHP diagnosis was associated with increased risks of accessing long-term sick leave and disability pension. The main diagnostic reason for this additional risk was the AHP diagnosis itself, not other comorbidites. HCC was the only specific underlying cause of death more common in AHP than in the general population but, we found no evidence of an overall increased risk of a premature death.

In a population-based study of 356 persons with AIP in Northern Sweden, Bylesjo et al. [1] found 20% of the subjects with symptomatic AIP reported accessing long-term sick leave or disability pension at a mean age of 45 years of age. We found 79% of those who self-reported being hospitalised for an acute attack accessed long-term sick leave and 46% accessed disability leave. Despite similarities between Norwegian and Swedish social benefit systems, it remains difficult to directly compare the results given differences in policies and registration practices [23], as well as different criteria for the definitions of symptomatic AHP and long-term sick leave between the two studies. In our study, the median age of 46 years for accessing disability pension was substantially younger than the general population (21 years difference). This finding suggests that symptomatic AHP results in long-term disability that is associated with a drastic loss in working years.

In the Norwegian Porphyria Registry, half the participants with AHP reported having chronic symptoms for more than one month, such as fatigue, stomach pain, and muscle weakness, which they ascribed to their AHP. Such findings are also commonly reported in many qualitative and cross-sectional studies in patients who have recurrent acute attacks [10, 12, 13]. However, such studies lack sufficient control groups. In our registry-based cohort study which compared persons with AHP to the entire population, we found many long-term sick leave episodes due to weakness/tiredness (8%), abdominal pain (3%), muscle/joint pain (4%), acute stress reaction (11%) and feeling depressed/depressive disorder (20%). However, such complaints were also common in the general population, and we found no evidence that persons with AHP had a comparatively increased risk of long-term sick leave due to these reasons. In our study, AHP was the most prevalent cause of long-term sick leave or for accessing disability pension.

We found an 84-fold excess risk of mortality due to HCC compared to the general population. The risk ratio was reduced to 54 in a crude hypothetical sensitivity analysis. We have previously described this risk [18], which has also been reported in other population based studies from several other countries [18]. An excess risk of death due to renal impairment has also been described in AHP. Andersson and Lithner [16] found that renal failure was the cause in 9% of AIP deaths between 1978 and 1990 in Northern Sweden. In line with this, we found that renal failure was cited as the underlying cause of death in two persons (4% of deaths) and as a contributing cause in five persons (9% of deaths) out of 55 deaths in total.

In our study, only two deaths were attributed to AHP itself, accounting for 8% of 24 deaths in persons who reported having symptomatic AHP. In a study by Linet et al. [24] conducted in Sweden (1977 and 1993) and Denmark (1965–1989), 41% of deaths were due to AIP. It is likely that developments in diagnosis, treatment, and follow-up have improved survival for persons with AHP and, therefore, this drop in mortality due to AHP is expected in our more recent study.

Linet et al. [24] reported a 1.9-fold overall increased risk of premature death in AIP patients due to cancer and ischemic heart disease (excluding AIP as a cause of death). Initially, we found a 1.3-fold increased risk of premature death but isolated to the unclassified sub-group, who were overrepresented by persons included in our study due to their deceased status. In a sensitivity analysis crude sensitivity analysis using all known persons with an AHP diagnosis across Norway which did not support our initial finding, and despite an increased risk of death due to HCC, we found no evidence of overall increased risk of premature death in AHP.

Strengths of the current study included the prospective population-based cohort design with a long follow-up period. Information regarding the outcomes was drawn from compulsory national registries and databases. We used the entire adult Norwegian population as our reference group and were able to adjust for potential confounders like age, sex, and educational attainment, as a proxy of socioeconomic status. However, residual confounding cannot be excluded, though we find it reassuring that results from the matched case-control and cohort analyses were very similar.

A limitation of this study was that we were unable to include all persons with AHP in Norway, as well as all asymptomatic AHP gene mutation carriers. Participation in the study was by consent, and persons who did not participate may differ to those who did. However, the consent rate was relatively high at 78%, negating this bias to an extent. We were also able to conduct a sensitivity analysis to re-assess our risk ratio estimates for all-cause mortality and death from HCC. The number of asymptomatic AHP gene mutation carriers is underrepresented in the current study, as predictive testing for AHP is voluntary and regulated by law in Norway. Another limitation was our inability to classify some persons with a verified AHP diagnosis as symptomatic or asymptomatic because they had not responded to clinical questionnaires. We were also dependent on self-reported clinical data to define AHP sub-groups, which may have resulted in some level of miss-classification. However, biochemical data provided some objective support to these otherwise self-selected groups, with 79% of the persons reporting having been hospitalised for an acute attack having at some point had a PBG four times the laboratory upper reference limit, compared to 24% of asymptomatic AHP gene mutation carriers. Finally, although long-term sick leave and disability pension are available for the entire population and include both persons consulted as inpatients and outpatients, the data are primarily used for administrative purposes and, therefore, the diagnostic accuracy of the data have not been validated. The underlying diagnostic codes from the Cause of Death Registry of Norway, on the other hand, has been shown to have good validity [22].

## Conclusion

In summary, we demonstrated in a large population-based cohort study that persons with symptomatic AHP were at increased risk of accessing long-term sick leave and disability pension due to their porphyria. This risk increased with AHP severity, with persons hospitalised at least once for an acute attack having the highest risk and asymptomatic gene mutation carriers having no additional risk. Although the risk of dying from HCC was found to be increased in symptomatic AHP, we found no evidence of overall increased risk of premature death in persons with symptomatic or asymptomatic AHP.

## Supplementary information

**Additional file 1: Table S1.** Matched analyses with 10 controls to each case, frequency-matched on age, sex and educational obtainment for each analysis.

## Data Availability

The data that support the findings of this study are available from Statistics Norway but restrictions apply to the availability of these data, which were used under license for the current study, and so are not publicly available. Data are however available from the authors upon reasonable request and with permission of Statistics Norway.

## References

[1] Bylesjo I, Wikberg A, Andersson C (2009). Clinical aspects of acute intermittent porphyria in northern Sweden: a population-based study. Scand J Clin Lab Invest.

[2] Jeans JB, Savik K, Gross CR, Weimer MK, Bossenmaier IC, Pierach CA (1996). Mortality in patients with acute intermittent porphyria requiring hospitalization: a United States case series. Am J Med Genet.

[3] Strand LJ, Felsher BF, Redeker AG, Marver HS (1970). Heme biosynthesis in intermittent acute prophyria: decreased hepatic conversion of porphobilinogen to porphyrins and increased delta aminolevulinic acid synthetase activity. Proc Natl Acad Sci U S A.

[4] Pischik E, Kauppinen R (2015). An update of clinical management of acute intermittent porphyria. Appl Clin Genet.

[5] Mykletun M, Aarsand AK, Stole E, Villanger JH, Tollanes MC, Baravelli C, et al. Porphyrias in Norway. Tidsskr Nor Laegeforen. 2014;134(8):831–6. 10.4045/tidsskr.13.0649 24780981

[6] Elder G, Harper P, Badminton M, Sandberg S, Deybach JC (2013). The incidence of inherited porphyrias in Europe. J Inherit Metab Dis.

[7] Chen B, Solis-Villa C, Hakenberg J, Qiao W, Srinivasan RR, Yasuda M (2016). Acute intermittent Porphyria: predicted pathogenicity of HMBS variants indicates extremely low penetrance of the autosomal dominant disease. Hum Mutat.

[8] Yang J, Zhu T, Zhao Y, Yu X, Zhu H, Jiang Y (2018). Acute intermittent Porphyria in the north of China: the acute attack effect on quality of life and psychological condition. Biomed Res Int.

[9] Millward LM, Kelly P, Deacon A, Senior V, Peters TJ (2001). Self-rated psychosocial consequences and quality of life in the acute porphyrias. J Inherit Metab Dis.

[10] Naik H, Stoecker M, Sanderson SC, Balwani M, Desnick RJ (2016). Experiences and concerns of patients with recurrent attacks of acute hepatic porphyria: a qualitative study. Mol Genet Metab.

[11] Gouya L, Bloomer J, Balwani M, Bissell DM, Rees D, Stölze U (2018). A prospective, multinational natural history study of patients with acute hepatic porphyria with recurrent attacks. J Hepatol.

[12] Simon A, Pompilus F, Querbes W, Wei A, Strzok S, Penz C (2018). Patient perspective on acute intermittent Porphyria with frequent attacks: a disease with intermittent and chronic manifestations. Patient..

[13] Gouya L, Ventura P, Balwani M, Bissell DM, Rees DC, Stolzel U, et al. EXPLORE: A Prospective, Multinational, Natural History Study of Patients with Acute Hepatic Porphyria with Recurrent Attacks. Hepatol. 2019. 10.1002/hep.30936 PMC7255459 31512765

[14] Neeleman RA, Wagenmakers M, Koole-Lesuis RH, Mijnhout GS, Wilson JHP, Friesema ECH (2018). Medical and financial burden of acute intermittent porphyria. J Inherit Metab Dis.

[15] Pallet N, Mami I, Schmitt C, Karim Z, Francois A, Rabant M (2015). High prevalence of and potential mechanisms for chronic kidney disease in patients with acute intermittent porphyria. Kidney Int.

[16] Andersson C, Lithner F (1994). Hypertension and renal disease in patients with acute intermittent porphyria. J Intern Med.

[17] Innala E, Andersson C (2011). Screening for hepatocellular carcinoma in acute intermittent porphyria: a 15-year follow-up in northern Sweden. J Intern Med.

[18] Baravelli CM, Sandberg S, Aarsand AK, Nilsen RM, Tollanes MC (2017). Acute hepatic porphyria and cancer risk: a nationwide cohort study. J Intern Med.

[19] Norsk porfyriregister | Nasjonalt servicemiljø for medisinske kvalitetsregistre https://www.kvalitetsregistre.no/registers/norsk-porfyriregister [.

[20] The Norwegian Tax Administration. 2019 [Available from: https://www.skatteetaten.no/en/person/national-registry/.

[21] Statistics Norway. Forløpsdatabasen-Trygd 2002 [Available from: https://www.ssb.no/sosiale-forhold-og-kriminalitet/artikler-og-publikasjoner/forlopsdatabasen-trygd.

[22] Pedersen AG, Ellingsen CL (2015). Data quality in the causes of death registry. Tidsskr Norske Laege.

[23] Thorsen SV, Friborg C, Lundstrøm B, Kausto J, Örnelius K, Sundell T (2015). Sickness absence in the Nordic countries.

[24] Linet MS, Gridley G, Nyren O, Mellemkjaer L, Olsen JH, Keehn S (1999). Primary liver cancer, other malignancies, and mortality risks following porphyria: a cohort study in Denmark and Sweden. Am J Epidemiol.

