# Supplementary material for: Sick leave, disability, and mortality in acute hepatic porphyria: a nationwide cohort study
Source: Orphanet J Rare Dis. 2020 Feb 21;15:56. doi: 10.1186/s13023-019-1273-4 (PMC7035738; doi:10.1186/s13023-019-1273-4)
Supplement: Supplementary file 1 — Additional file 1: Table S1. Matched analyses with 10 controls to each case, frequency-matched on age, sex and educational obtainment for each analysis. [file 13023_2019_1273_MOESM1_ESM.docx]

Supplementary Table 1. Matched analyses with 10 controls to each case, frequency-matched on age, sex and educational obtainment for each analysis

|  | **Cases / No. at risk** | **Median age at event (IQR)** | **Annual rate per 100 (95% CI)** | **Hazard ratios (95% CI)** |
| --- | --- | --- | --- | --- |
| **Long-term sick leave** |  |  |  |  |
| **Matched controls** | 1799 / 2930 | 31 (25, 40) | 6.3 (6.0, 6.6) | 1.0 |
| **AHP (all groups)** | 205 / 293 | 28 (23, 34) | 9.5 (8.3, 10.9) | 1.6 (1.3, 1.8) |
| **Sub-groups:** |  |  |  |  |
| Matched controls | 267/ 420 | 31 (26, 38) | 6.5 (5.8, 7.3) | 1.0 |
| Hospitalised | 33 / 42 | 24 (22, 28) | 13.8 (9.8, 19.3) | 2.2 (1.6, 3.2) |
| Matched controls | 692 / 1,039 | 32 (25, 41) | 6.4 (5.9, 6.9) | 1.0 |
| Non-hospitalised | 86 / 104 | 27 (23, 33) | 11.8 (9.5, 14.6) | 1.9 (1.5, 2.3) |
| Matched controls | 583/ 1,031 | 30 (25, 41) | 5.9 (5.4, 6.3) | 1.0 |
| Asymptomatic | 52 / 103 | 33 (27, 44) | 5.7 (4.4, 7.5) | 1.0 (0.8, 1.4) |
| Matched controls | 298 / 460 | 29 (24, 40) | 7.0 (6.3, 7.9) | 1.0 |
| Unclassified | 34 / 46 | 22 (21, 31) | 10.4 (7.5, 14.6) | 1.5 (1.1, 2.2) |
| **Disability pension** |  |  |  |  |
| **Reference** | 460 / 2,900 | 67 (59, 67) | 0.9 (0.8, 1.0) | 1.0 |
| **AHP (all gropus)** | 82 / 290 | 60 (47, 67) | 1.8 (1.4, 2.2) | 2.0 (1.6-2.5) |
| **Sub-groups:** |  |  |  |  |
| Matched controls | 29 / 410 | 67 (58, 67) | 0.9 (0.8, 1.0) | 1.0 |
| Hospitalised | 19 / 41 | 46 (38, 58) | 3.0 (1.9, 4.7) | 4.0 (2.4, 6.8) |
| Matched controls | 208 / 1040 | 67 (59, 67) | 1.1 (1.0, 1.3) | 1.0 |
| Non-hospitalised | 34 / 104 | 61 (51, 67) | 1.8 (1.3, 2.6) | 1.8 (1.2, 2.6) |
| Matched controls | 121 / 1000 | 67 (60, 67) | 0.7 (0.6, 0.9) | 1.0 |
| Asymptomatic | 16 / 100 | 62.4 (54, 67) | 1.1 (0.6, 1.7) | 1.5 (0.9, 2.6) |
| Matched controls | 96 / 450 | 67 (57, 67) | 1.2 (1.0, 1.5) | 1.0 |
| Unclassified | 13 / 45 | 61 (39, 67) | 1.9 (1.1, 3.4) | 1.5 (0.9, 2.7) |
| **Mortality** |  |  |  |  |
| **Reference** | 412 / 3,300 | 84 (76, 91) | 0.7 (0.6, 0.8) | 1.0 |
| **AHP (all groups)** | 55 / 330 | 80 (73, 87) | 1.0 (0.7, 1.2) | 1.4 (1.1, 1.9) |
| **Sub-groups:** |  |  |  |  |
| Matched controls | 47 / 470 | 83 (76, 92) | 0.5 (0.4, 0.7) | 1.0 |
| Hospitalised AHP | 6 / 47 | 78 (74, 90) | 0.7 (0.3, 1.5) | 1.1 (0.5, 2.7) |
| Matched controls | 160 / 1200 | 85 (76, 93) | 0.7 (0.6, 0.9) | 1.0 |
| Non-hospitalised | 18 / 120 | 83 (75, 87) | 0.7 (0.5, 1.2) | 1.2 (0.7-1.9) |
| Matched controls | 74 / 996 | 85 (76, 91) | 0.4 (0.4, 0.6) | 1.0 |
| Asymptomatic | 7 / 106 | 84 (76, 90) | 0.4 (0.2, 0.8) | 0.9 (0.4, 0.9) |
| Matched controls | 118 / 569 | 83 (76, 92) | 1.2 (1.0, 1.4) | 1.0 |
| Unclassified | 24 /57 | 78 (74, 90) | 3.0 (2.0, 4.5) | 2.9 (1.9, 4.6) |

Note: HR: CI: Confidence intervals; IQR: interquartile range (25th, 75th percentiles)
